# Strength of the [Z–I···Hal]^−^ and [Z–Hal···I]^−^ Halogen Bonds: Electron Density Properties and Halogen Bond Length as Estimators of Interaction Energy

**DOI:** 10.3390/molecules26072083

**Published:** 2021-04-05

**Authors:** Maxim L. Kuznetsov

**Affiliations:** 1 Centro de Química Estrutural, Instituto Superior Técnico, Universidade de Lisboa, Avenida Rovisco Pais, 1049-001 Lisbon, Portugal; max@mail.ist.utl.pt; Tel.: +351-218-419-236; 2 Institute of Chemistry, Saint Petersburg State University, Universitetskaya Nab. 7/9, 199034 Saint Petersburg, Russia

**Keywords:** bond critical point properties, interaction energy, bond energy, bond strength, density functional theory, electron density, energy density, halogen bond, QTAIM

## Abstract

Bond energy is the main characteristic of chemical bonds in general and of non-covalent interactions in particular. Simple methods of express estimates of the interaction energy, E_int_, using relationships between E_int_ and a property which is easily accessible from experiment is of great importance for the characterization of non-covalent interactions. In this work, practically important relationships between E_int_ and electron density, its Laplacian, curvature, potential, kinetic, and total energy densities at the bond critical point as well as bond length were derived for the structures of the [Z–I···Hal]^−^ and [Z–Hal···I]^−^ types bearing halogen bonds and involving iodine as interacting atom(s) (totally 412 structures). The mean absolute deviations for the correlations found were 2.06–4.76 kcal/mol.

## 1. Introduction

The halogen bond is one of the most important types of non-covalent interactions being second only to hydrogen bonds in its significance. According to the IUPAC definition, “a halogen bond occurs when there is evidence of a net attractive interaction between an electrophilic region associated with a halogen atom in a molecular entity and a nucleophilic region in another, or the same, molecular entity” [1]. Weak interactions in general and halogen bonds in particular underly the self-assembly strategy [2,3,4] leading to new functional materials [5,6,7,8,9,10,11,12,13,14,15,16,17] and are applied in crystal engineering [15], catalysis [18,19,20], and drug design [21,22,23].

The supramolecular and cluster chemistry of halide anions currently attracts much attention [24,25,26,27,28,29] due to their important role in chemistry and biology [30,31,32,33]. Halogen bonds formed by a halide anion belong to a particularly interesting type of non-covalent interactions. They play a key role in the design of chemical receptors for anion recognition [27,34,35,36,37,38], in the anion-assisted asymmetric catalysis [39,40,41,42], and in the development of approaches toward new materials bearing useful electronic properties [43,44,45,46,47,48,49,50].

Properties of functional materials and supramolecular structures formed by halogen bonding are controlled by its strength. Therefore, halogen bond energy is the most important characteristic of these interactions. However, determination of the bond energy (E_b_) is a difficult task. Experimental methods (e.g., mass spectrometry, vibrational or NMR spectroscopy, calorimetry, vapor density measurements) are usually associated with complex technical procedures and can be applied to a very limited number of structures. The direct theoretical calculations of E_b_ for the A···B bond as the energy difference between the structure A···B and the isolated molecules A and B may be successfully applied to intermolecular non-covalent interactions in the gas phase. However, in the condensed phase, molecules are usually bound with each other by a network of several non-covalent interactions, and an adequate fragmentation which affects only the bond of interest is often impossible. In such a situation, an approximation of E_b_ through other parameters easily accessible from experiment becomes very important [51,52,53,54,55,56,57,58,59,60]. Among these parameters, bond distance [61,62,63,64,65,66,67,68,69,70,71,72,73,74,75,76,77,78], electron density properties [61,79,80,81,82,83,84,85,86,87,88,89,90,91,92,93,94,95,96,97,98,99,100,101,102,103,104,105,106,107,108,109,110,111,112,113], and force constants [57,73,114,115] are the most common. In fact, the application of E_b_-property relationships is often the only possibility to estimate the energy of non-covalent interactions in the condensed phase.

A broad practical utilization of the E_b_-property relationships for the estimates of E_b_ started with a publication by Espinosa, Molins, and Lecomte [82], where a simple correlation between the bond energy and the potential energy density (V_b_) at the bond critical point (BCP) was obtained for the X–H···O hydrogen bonds (X = C, N, O):

E_int_ ~ 0.5V_b_

where E_int_ is the interaction energy of the A···B bond defined as E_int_ = E_A···B_ − E_A_ − E_B_ (A and B are fragments with unrelaxed geometry corresponding to the structure A···B). Later, several other equations for the approximation of E_int_ through the electron density properties were published for hydrogen and halogen bonds (Table 1 and Table S1). Meanwhile, it was shown that these relationships are not universal and may be applied only to the type of interaction for which they were derived [116,117,118].

Recently, the author started a project to establish practically useful correlations between the interaction energy and properties which could be easily determined from experiment for halogen bonds of various types, including those formed by a halide anion. In the previous work [116], such relationships were found for structures of the [(A)*_n_*Z–Y···X]^−^ type (X, Y = F, Cl, Br; Z = F, Cl, Br, C, N, O, H, S, P, Si, B; 441 structures in total). The aim of this work was to include in the consideration the structures bearing the interacting iodine atom(s), thus completing the whole series of halogen bonds with a halide anion. In this work, structures of the [(A)*_n_*Z–I···X]^−^ and [(A)*_n_*Z–Y···I]^−^ types (X = F, Cl, Br, I; Y = Cl, Br, I; Z = F, Cl, Br, I, C, N, O, H, S, P, Si, B) were calculated. The correlations between E_int_ and the electron density (ρ_b_), its Laplacian (∇^2^ρ_b_), the curvature of ρ(r) which is parallel to the bond path direction (positive) (λ_||,b_), the potential, kinetic, and total energy densities (V_b_, G_b_, and H_b_) at BCP, and the halogen bond length (d_Y···X_) were established.

Two points should be mentioned here. First, the interactions with the iodine atom as a halogen bond donor are the most important among all halogen bonds since the existence of a prominent σ-hole at the iodine atom provides particularly high stability of these interactions. Second, reliable relationships can be obtained only for sufficiently large sets of the structures [(A)*_n_*Z–Y···X]^−^ which include different types of second-order atoms Z and third-order groups A, otherwise, the relationships found may suffer a significant overfitting. Therefore, a large statistically significant set of 412 structures bearing twelve different types of the Z atom was used in this work.

## 2. Computational Details

Full geometry optimization of the main set of structures was carried out at the density functional theory (DFT) level by using the M06-2X functional [121] with the help of the Gaussian 09 [122] program package applying tight optimization criteria and an ultrafine integration grid. Cartesian d and f basis functions (6d, 10f) were used in all calculations. The ADZP basis set taken from the Basis Set Exchange library [123] was applied for geometry optimization. The energies of the equilibrium structures were refined by the second-order scalar relativistic correction using the Douglas−Kroll−Hess method (DKH) and single-point calculations with the ADZP–DKH basis set [123,124]. The ADZP–DKH basis set was constructed from the DZP–DKH basis set [125,126,127] by addition of diffuse functions taken from the ADZP basis set.

Similar level of theory was applied in the previous work for the analysis of correlations for the [(A)*_n_*Z–Y···X]^−^ structures (X, Y = F, Cl, Br) [116]. It was shown by Kozuch and Martin [128] for a set of 51 structures bearing halogen bonds that the interaction energies calculated with the M06-2X functional correlate well with the reference CCSD(T) level providing the root-mean-square deviation, the mean signed error, and the maximum error of 0.43, 0.01, and 1.58 kcal/mol, respectively. It was also shown [116] that the E_int_(V_b_) correlations for the [(A)*_n_*Z–Y···X]^−^ structures (X, Y = F, Cl, Br) obtained at the M06-2X level of theory have similar parameters as those for the MP4, CCSD, and CCSD(T) methods. On the other hand, it was found [116] that the simple double-zeta quality basis set 6-31+G* which is similar to ADZP provides the interaction energy values and parameters of the E_int_(V_b_) correlations close to those obtained at the much more extended triple-zeta basis set 6-311++G(3df,3pd).

The applicability of the M06-2X/ADZP–DKH level of theory for the reproduction of experimental interaction energies and electron densities is additionally discussed below in the Results. For this analysis, the interaction energies and electron density values were calculated at the CCSD, CCSD(T), and PBE0-D3BJ levels of theory. The ADZP–DKH, 6-31+G*, 6-311+G**, and aug-cc-pVTZ basis sets were applied.

The Hessian matrix was calculated for all optimized structures to prove the location of correct minima. No symmetry operations were applied during the calculations. The stability test was performed, and stable solutions were achieved for all structures using the keyword STABLE(OPT).

The interaction energies for the Y…X bond in [(A)*_n_*Z–Y···X]^−^ were calculated using Equation (1):

E_int_ = E([(A)*_n_*Z–Y···X]^−^)_ADZP–DKH_ − E((A)*_n_*Z–Y)_ADZP–DKH_ − E(X^−^)_ADZP–DKH_ + BSSE_ADZP_ (1)

where BSSE is a basis set superposition error estimated using the counterpoise (CP) method [129,130] with the ADZP basis set. Geometries of the (A)*_n_*Z–Y fragments were unrelaxed and corresponded to those in [(A)*_n_*Z–Y···X]^−^. The topological analysis of the electron density distribution was performed with the help of the Atoms in Molecules (AIM) method developed by Bader [131] using the AIMAll program [132].

## 3. Computational Models

Structures of the [(A)*_n_*Z–I···X]^−^ and [(A)*_n_*Z–Y···I]^−^ types (X = F, Cl, Br, I; Y = Cl, Br, I; Z = F, Cl, Br, I, C, N, O, H, S, P, Si, B; 412 structures in total) were selected for the calculations. Among them, 70 structures were in each series [(A)*_n_*Z–I···X]^−^ with X = F, Cl, or Br and [(A)*_n_*Z–Br···I]^−^, 65 structures were in the [(A)*_n_*Z–I···I]^−^ series, and 67 structures were in the [(A)*_n_*Z–Cl···I]^−^ series. For Z = C and N, different orbital hybridizations of these atoms were considered. For Z = S and P, different oxidation states of these atoms were considered. The groups A (totally 66 groups) vary from the electron donor to the electron acceptor ones providing a broad span of interaction energies for the halogen bond. The complete list of the calculated structures is given in Table S2.

The M06-2X/ADZP–DKH method was validated toward the reproduction of the experimental electron densities for the set of eleven structures bearing halogen bonds of different types (I…I, Cl…Cl, Br…Cl, Cl…O, Br…Br, I…N, and I…O) and for which the experimental electron densities are known. Performance of the M06-2X and PBE0-D3BJ functionals was also compared for the set of ten structures of the [(A)*_n_*Z–I···F]^−^ type ([Br–I…F]^−^, [H–I…F]^−^, [(F_3_C)O_2_S–I…F]^−^, [F_3_Si–I…F]^−^, [*p*-H_2_N-C_6_H_4_-Br–I…F]^−^, [Me_3_Si–I…F]^−^, [MeO–I…F]^−^, [(Me)O_2_S–I…F]^−^, [Ph–I…F]^−^, and [(*p*-Me-C_6_H_4_)_2_N–I…F]^−^), which provide the E_int_ span from –9 to –73 kcal/mol.

## 4. Results

### 4.1. Test of the Computational Method

Since the main goal of this work is to establish relationships between the interaction energy and the experimentally accessible properties such as electron density and derivatives and the length of the halogen bond, it is essential, first, to test the ability of the computational method used to reasonably reproduce these experimental properties. Results of such analysis are presented in this section.

**Interaction energy.** It is a usual practice to test reliability of a computational method for the estimates of interaction energies against a reference level of theory, such as coupled cluster (CC) approaches. Meanwhile, even the CCSD(T) level often considered as the “gold standard” may be associated with some degree of uncertainty, for instance, due to issues related with the basis set superposition error and its correction [133]. Therefore, when it is possible, experimental data should always be considered as the ultimate reference for method benchmarking.

Here, interaction energies were calculated for several structures bearing the hydrogen or halogen bonds with a halide anion for which experimental data are available (i.e., H_3_C–H···F^−^, [F–H–F]^−^, HO–H···Cl^−^, HO–H···I^−^, H_3_C–H···I^−^, and [I–I–I]^−^). As can be seen in Table 2, the M06-2X functional with the moderate double-zeta quality basis sets 6-31+G* or ADZP–DKH and the CP correction provide good agreement with the experimental bond energies with the mean absolute deviation (MAD) of 1.2 kcal/mol for the whole set of these structures and of 0.6 kcal/mol excluding [F–H–F]^−^. The experimental error is in the range of 0.1–1.6 kcal/mol, and theoretical M06-2X values fall within the 3σ interval for four of six structures. The interaction energies calculated at the CCSD and CCSD(T) levels were strongly underestimated when the CP correction was applied. The CCSD method performed worse than M06-2X for H_3_C–H···F^−^ and HO–H···Cl^−^ even with the aug-cc-pVTZ basis set, and only the CCSD(T) level with the triple-zeta basis set and without the CP correction provided better results than the DFT approach (MAD = 0.3 kcal/mol). Thus, considering that typical MAD values for the E_int_ property relationships found for the structures [(A)*_n_*Z–Hal_1_···Hal_2_]^−^ are 2–4 kcal/mol (see [116] and results of this work below), the M06-2X/6-31+G*(or ADZP–DKH) level of theory is reliable for the analysis of interaction energies.

**Electron density**. Recently, it has been shown that the M06-2X functional is not sufficiently good to reproduce the experimental electron density properties for isolated atoms and atomic cations [138]. Here, the ability of this functional to reproduce the experimental ρ_b_ values was tested for the BCPs corresponding to the halogen bonds in eleven structures (Table 3). The calculated structures corresponded to the experimental X-ray ones. The results indicate that the M06-2X/ADZP–DKH method adequately reproduces the experimental electron density at BCPs in these structures with the MAD and RMSD values of 0.006 and 0.009 e/A^3^, respectively. The slope of the correlation ρ_b,theor_ against the experimental values ρ_b,exp_ was quite close to unity (1.07, Figure S1). The PBE0 functional found by Medvedev et al. [138] as one of the best to reproduce the electron density properties in isolated atoms and atomic cations demonstrates the performance similar to M06-2X for halogen bonds (MAD = 0.006 e/A^3^, RMSD = 0.009 e/A^3^, slope, 1.12).

Correspondingly, the E_int_(V_b_) relationships obtained for the small set of ten structures of the [(A)*_n_*Z–I···F]^−^ type were similar for both M06-2X and PBE0-D3BJ functionals (Figure S2). The deviation of E_int_ estimated by these two methods was 2.27 kcal/mol at V_b_ = 20 kcal/(mol•bohr^3^) and it was 3.96 kcal/mol at V_b_ = 100 kcal/(mol•bohr^3^). These deviations were lower than MAD for the whole set of seventy [(A)*_n_*Z–I···F]^−^ structures (4.76 kcal/mol, see below). All these results indicate that the relationships found in this work at the M06-2X/ADZP–DKH level are reliable for the determination of E_int_ using experimental electron density properties.

### 4.2. Interaction Energies

After validation of the computational method, the interaction energies calculated for 412 structures of the [(A)*_n_*Z–I···X]^−^ and [(A)*_n_*Z–Y···I]^−^ types and the corresponding E_int_ property relationships were discussed (in this and the following sections). The calculated BSSE-corrected interaction energies for the halogen bonds in these structures varied between 2.53 and −88.43 kcal/mol. These bonds were the strongest for the [(A)*_n_*Z–I···F^−^] series. The highest dispersion of E_int_ was found for the [(A)*_n_*Z–I···F]^−^ and [(A)*_n_*Z–Cl···I]^−^ series. The halogen bonds in the series [(A)*_n_*Z–I···Cl]^−^, [(A)*_n_*Z–I···Br]^−^, and [(A)*_n_*Z–I···I]^−^ have similar strengths and dispersion.

### 4.3. The E_int_(V_b_), E_int_(G_b_), and E_int_(ρ_b_) Relationships

**Figure 1:**
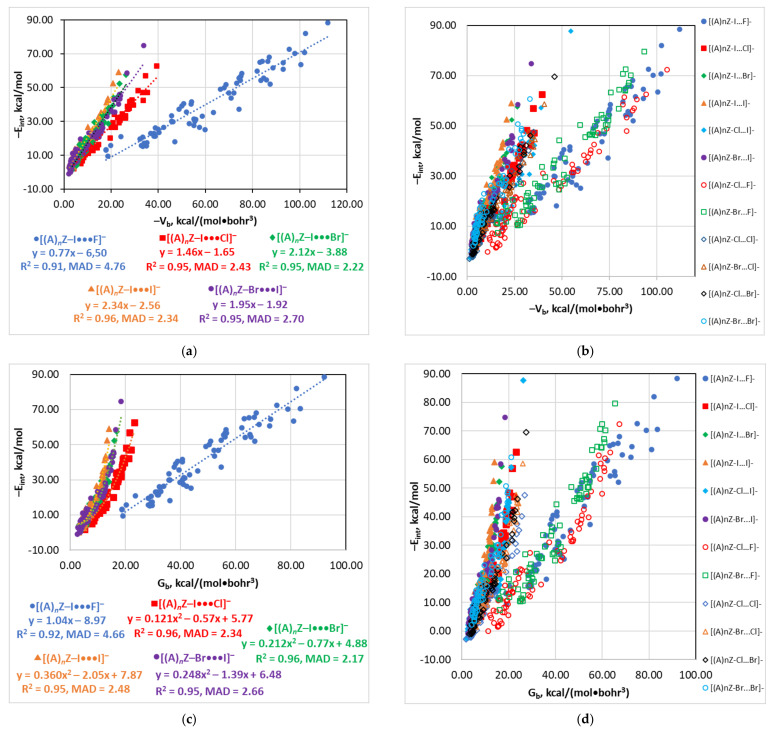

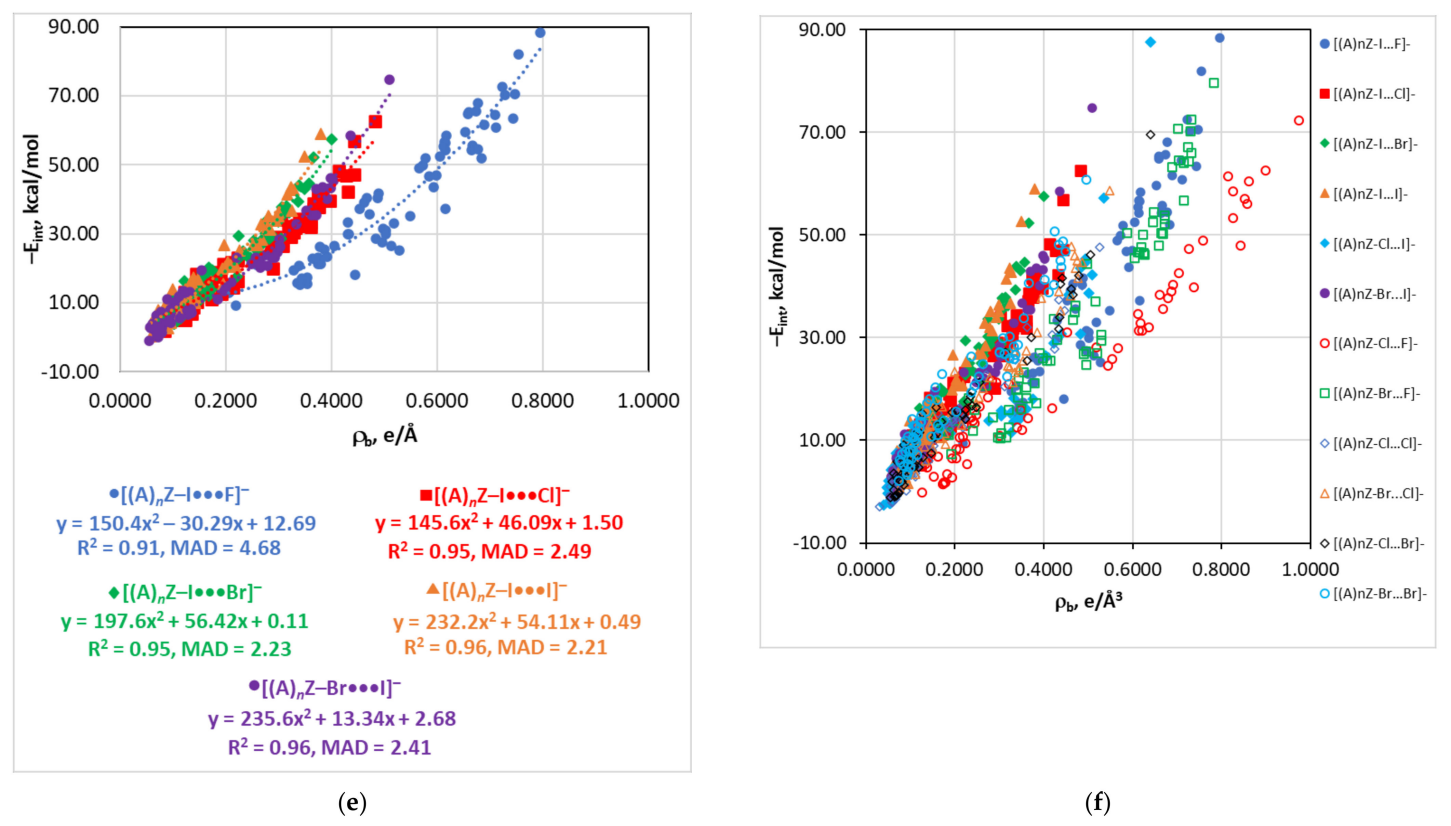
Plots of −E_int_ against −V_b_, G_b_, and ρ_b_ for the structures [(A)*_n_*Z–Y···I]^−^ and [(A)*_n_*Z–I···X]^−^ (**a**,**c**,**e**) and [(A)*_n_*Z–Y···X]^−^ (**b**,**d**,**f**); data for the non-iodine structures were taken from [116].

These types of relationships were recommended for the predictions of E_int_ for the hydrogen bonds X–H…O and FH…FR [82,83] and for the halogen bonds [(A)*_n_*Z–Y···X]^−^ (X, Y = F, Cl, Br) bearing halide anions [116]. Here, the linear correlations between E_int_ and V_b_ were found for all series except [(A)*_n_*Z–Cl···I]^−^ (Figure 1). The E_int_(G_b_) relationship was linear only for the [(A)*_n_*Z–I···F]^−^ series and it was approximated by a binominal function for [(A)*_n_*Z–I···X]^−^ (X = Cl, Br, or I) and [(A)*_n_*Z–Br···I]^−^. Finally, the E_int_(ρ_b_) dependence was quadratic for all series.

**Figure 2:**
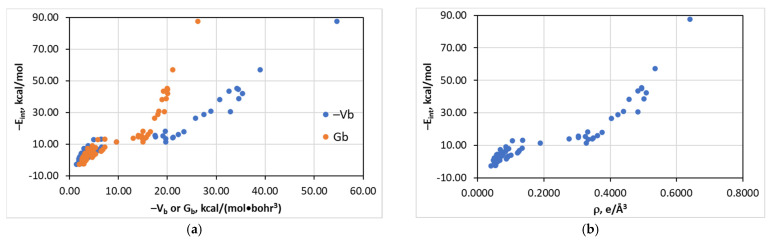
Plots of −E_int_ against −V_b_, G_b_ (**a**), and ρ_b_ (**b**) for the structures [(A)*_n_*Z–Cl···I]^−^.

An interesting relationship distinct from all other bonds with halide anions was obtained for the [(A)*_n_*Z–Cl···I]^−^ series (Figure 2). These structures could not be treated by a single linear or quadratic E_int_(V_b_), E_int_(G_b_), or E_int_(ρ_b_) function. Instead, the horizontal branch is clearly visible in the range of E_int_ of 10–20 kcal/mol. The interaction energy could be reasonably approximated by polynomial fourth-order functions, but such an approximation is usually associated with significant overfitting and cannot be recommended for practical use. The reasons of this peculiar situation are discussed below.

Analysis of the relationships obtained for the structures [(A)*_n_*Z–Y···X]^−^ in this and previous [116] works allows making the following additional conclusions and generalizations. First, all three estimators, V_b_, G_b_, and ρ_b_, behave similarly (Table 4). Second, correlations for the [(A)*_n_*Z–Y···F]^−^ series (R^2^ = 0.91–0.95, MAD = 3.29–4.76 kcal/mol) are of a lower quality compared to other series (R^2^ = 0.93–0.96, MAD = 2.06–3.01), except [(A)*_n_*Z–Cl···I]^−^. Third, the slope of the relationships is strongly affected by the nature of the Y and X atoms. For instance, for the V_b_ estimator, the lowest slope (0.69–0.84) and the highest negative intercept (−3.68 ÷ −6.50) were found for X = F. The highest slopes were detected for [(A)*_n_*Z–I···I]^−^ (2.34) as well as for the [(A)*_n_*Z–Y···X]^−^ (Y = Br, X = I; Y = I, X = Br, X = Y = Br) series (1.55–2.12). The series with (X or/and Y) = Cl but (X and Y) ≠ F exhibited an intermediate slope (1.26–1.46). Such a behavior can be qualitatively interpreted by the different properties of the halogen atoms forming the Y···X bond. First of all, the electronegativity of the X atom decreases along the series F > Cl > Br > I. There is a clear trend between the slope of the E_int_(V_b_) dependence and the difference of the electronegativities of X and Y (Figure S3). Thus, the higher the electronegativity difference, the lower the E_int_ at a given energy density at BCP. This conclusion becomes understandable if we recall that ionic bonds have high interaction energies but low electron densities at BCP. The second factor is an enhancement of polarizability, diffuse character of electron shells, and the role of dispersion forces from X = F to I. Electronic effects from the (A)*_n_*Z group on the Y···X bond are more pronounced if the electron shells of X are more diffuse, i.e., from X = F to I. Since the higher dispersion contribution and more diffuse electron shells are associated with lower concentration of electron or energy density at BCP, E_int_ values are higher for most electron acceptor groups (A)*_n_*Z at a given value of E_int_ in the case of heavier atom X.

Fourth, some series form distinct groups which can be approximated by a single relationship of a reasonable quality. There are three such groups when considering the E_int_(V_b_) dependence, i.e., (i) [(A)*_n_*Z–I···I]^−^ + [(A)*_n_*Z–I···Br]^−^ + [(A)*_n_*Z–Br···I]^−^, (ii) [(A)*_n_*Z–I···Cl]^−^ + [(A)*_n_*Z–Cl···Cl]^−^ + [(A)*_n_*Z–Br···Cl]^−^ + [(A)*_n_*Z–Cl···Br]^−^, and (iii) [(A)*_n_*Z–I···F]^−^ + [(A)*_n_*Z–Br···F]^−^ + [(A)*_n_*Z–Cl···F]^−^ groups. Each of them may be approximated by a linear function (R^2^ = 0.93–0.95, MAD = 2.58–4.45 kcal/mol). The E_int_(G_b_) and E_int_(ρ_b_) dependences are characterized by a higher dispersion between series. They are more sensitive to the nature of the Y and X atoms and, therefore, such a grouping is not efficient for those relationships.

Fifth, the relationships for any series of the [(A)*_n_*Z–Y···X]^−^ structures were significantly different from those obtained previously for the hydrogen bonds X–H…O and FH…FR [82,83] and for the neutral halogen bonds [117,119,120] (Table 1).

### 4.4. The E_int_(∇^2^ρ_b_) Relationship

**Figure 3:**
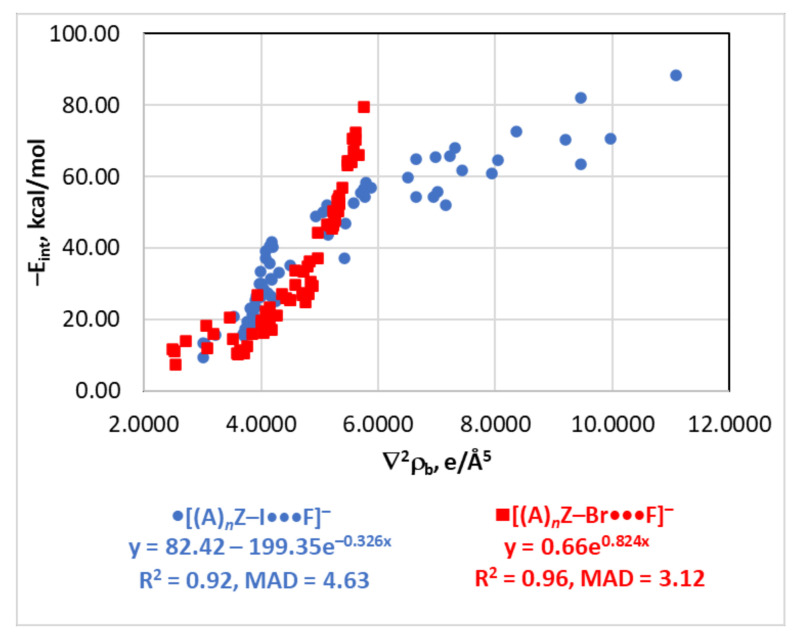
Plots of –E_int_ against ∇^2^ρ_b_ for the [(A)*_n_*Z–I···F]^−^ and [(A)*_n_*Z–Br···F]^−^ series (data for the [(A)*_n_*Z–Br···F]^−^ series were taken from [116]).

The Laplacian of electron density may be used as an estimator of E_int_ only for the [(A)*_n_*Z–I···F]^−^ (this work) and [(A)*_n_*Z–Br···F]^−^ [116] series (Figure 3). The relationships for these two series are exponential with the different sign of the exponents. The E_int_(∇^2^ρ_b_) functions for other series are not well-defined (Figure S4).

### 4.5. The E_int_(λ_||,b_) Relationship

**Figure 4:**
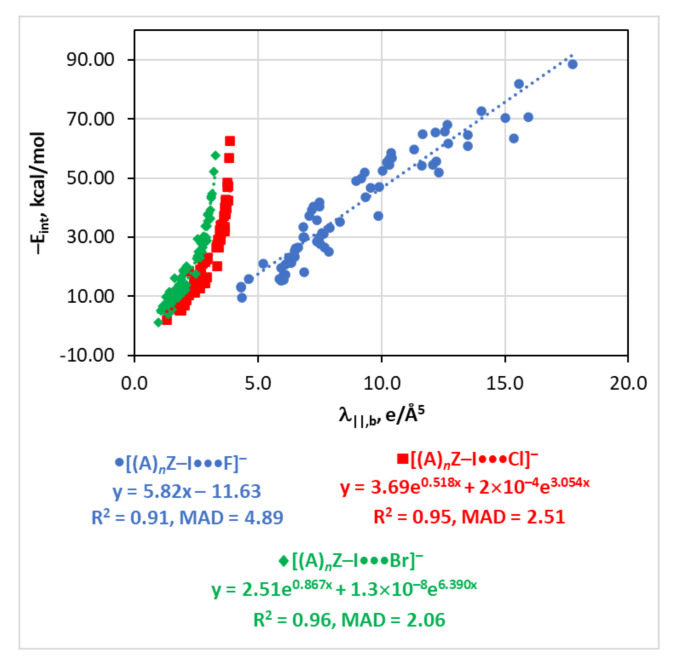
Plots of −E_int_ against λ_||,b_ for the [(A)*_n_*Z–I···X]^−^ series (X = F, Cl, Br).

The curvature of the electron density distribution can serve for the estimates of E_int_ only for three series calculated in this work, i.e., [(A)*_n_*Z–I···F]^−^, [(A)*_n_*Z–I···Cl]^−^, and [(A)*_n_*Z–I···Br]^−^ (Figure 4). The E_int_(λ_||,b_) relationship for the first of these series is linear and of a lower quality compared to the E_int_(V_b_), E_int_(G_b_), and E_int_(ρ_b_) functions. The last two series are very well approximated by two exponential functions (R^2^ = 0.95–0.96, MAD = 2.06–2.51 kcal/mol). For the series [(A)*_n_*Z–I···I]^−^ and [(A)*_n_*Z–Br···I]^−^, the λ_||,b_ parameter is not sensitive to determine E_int_ at high values (E_int_ > 30 kcal/mol) (Figure S5). The E_int_(λ_||,b_) fittings at lower E_int_ are also not reasonable. Finally, the E_int_(λ_||,b_) function is not well-defined for [(A)*_n_*Z–Cl···I]^−^.

### 4.6. The E_int_(H_b_) Relationship

**Figure 5:**
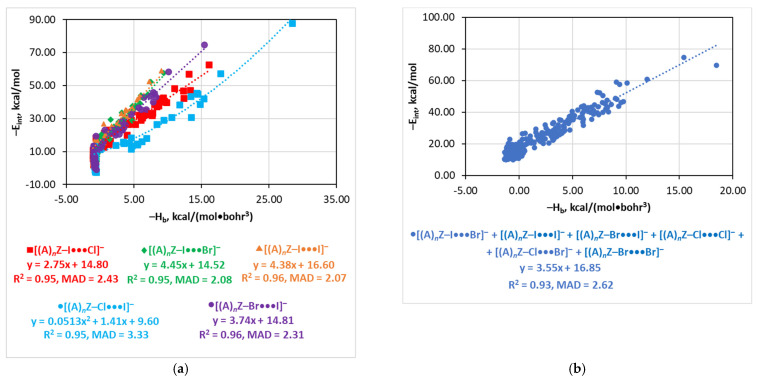
Plots of −E_int_ against −H_b_ for the individual [(A)*_n_*Z–Y···X]^−^ series (**a**) and for the combined set of the structures (**b**) (the fittings were performed for points corresponding to −E_int_ > 10 kcal/mol, data for the non-iodine structure were taken from [116]).

The quality of the total energy density as an estimator of E_int_ depends on the nature of the Y and X atoms. It cannot be used for the [(A)*_n_*Z–I···F]^−^ series due to high dispersion of the E_int_(H_b_) function at lower E_int_ and its low sensitivity at higher E_int_ (Figure S6). For other series bearing the iodine Y and/or X atom, the E_int_(H_b_) relationship is not sensitive for the weakest interactions with E_int_ < 10 kcal/mol. However, for stronger interactions, good linear dependences were observed (R^2^ = 0.95–0.96, MAD = 2.07–2.43 kcal/mol), except for the [(A)*_n_*Z–Cl···I]^−^ series which was approximated by a quadratic function (R^2^ = 0.95, MAD = 3.33 kcal/mol) (Figure 5). Six series (i.e., [(A)*_n_*Z–I···Br]^−^, [(A)*_n_*Z–I···I]^−^, [(A)*_n_*Z–Br···I]^−^, [(A)*_n_*Z–Cl···Cl]^−^, [(A)*_n_*Z–Cl···Br]^−^, and [(A)*_n_*Z–Br···Br]^−^) are described together by a single linear function of the reasonable quality (R^2^ = 0.93, MAD = 2.62 kcal/mol for E_int_ > 10 kcal/mol) (Figure 5).

### 4.7. The E_int_(d_Y…X_) Relationship

**Figure 6:**
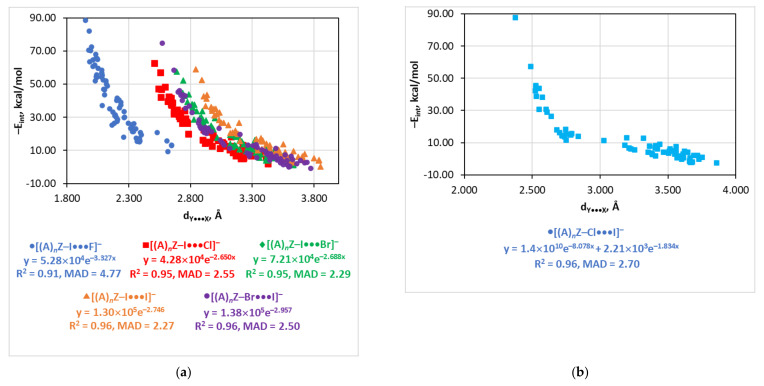
Plots of −E_int_ against d_Y···X_ for the [(A)*_n_*Z–I···X]^−^, [(A)*_n_*Z–Br···I]^−^ (**a**) and [(A)*_n_*Z–Cl···I]^−^ (**b**) series.

All series except for [(A)*_n_*Z–Cl···I]^−^ demonstrate reasonable exponential relationships between interaction energy and the length of the halogen bond (Figure 6). The quality of these relationships is similar to those with V_b_, G_b_, and ρ_b_. There are only two couples of series which can be approximated by single correlations, i.e., [(A)*_n_*Z–I···Br]^–^ + [(A)*_n_*Z–Br···I]^−^ and [(A)*_n_*Z–Br···Cl]^−^ + [(A)*_n_*Z–Cl···Br]^−^. For other series, the fitting parameters are quite different from each other. Similarly to the other estimators, the E_int_(d_Y…X_) dependence is complex for the [(A)*_n_*Z–Cl···I]^−^ series. There is a horizontal branch for the interval of E_int_ 5–15 kcal/mol. Meanwhile, in this case, the dependence may be quite reasonably approximated by a two-exponential function (R^2^ = 0.96, MAD = 2.70 kcal/mol).

### 4.8. The [(A)_n_Z–Cl···I]^−^ Series

**Figure 7:**
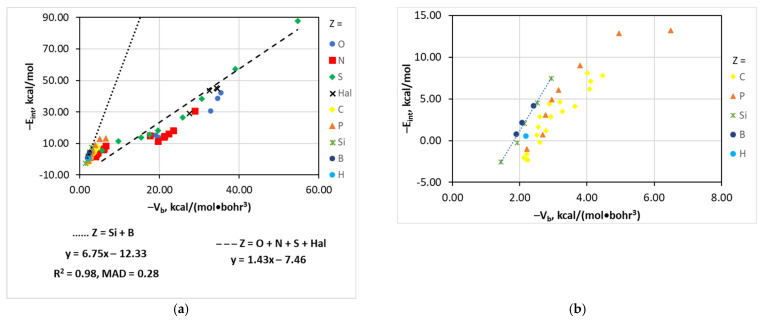

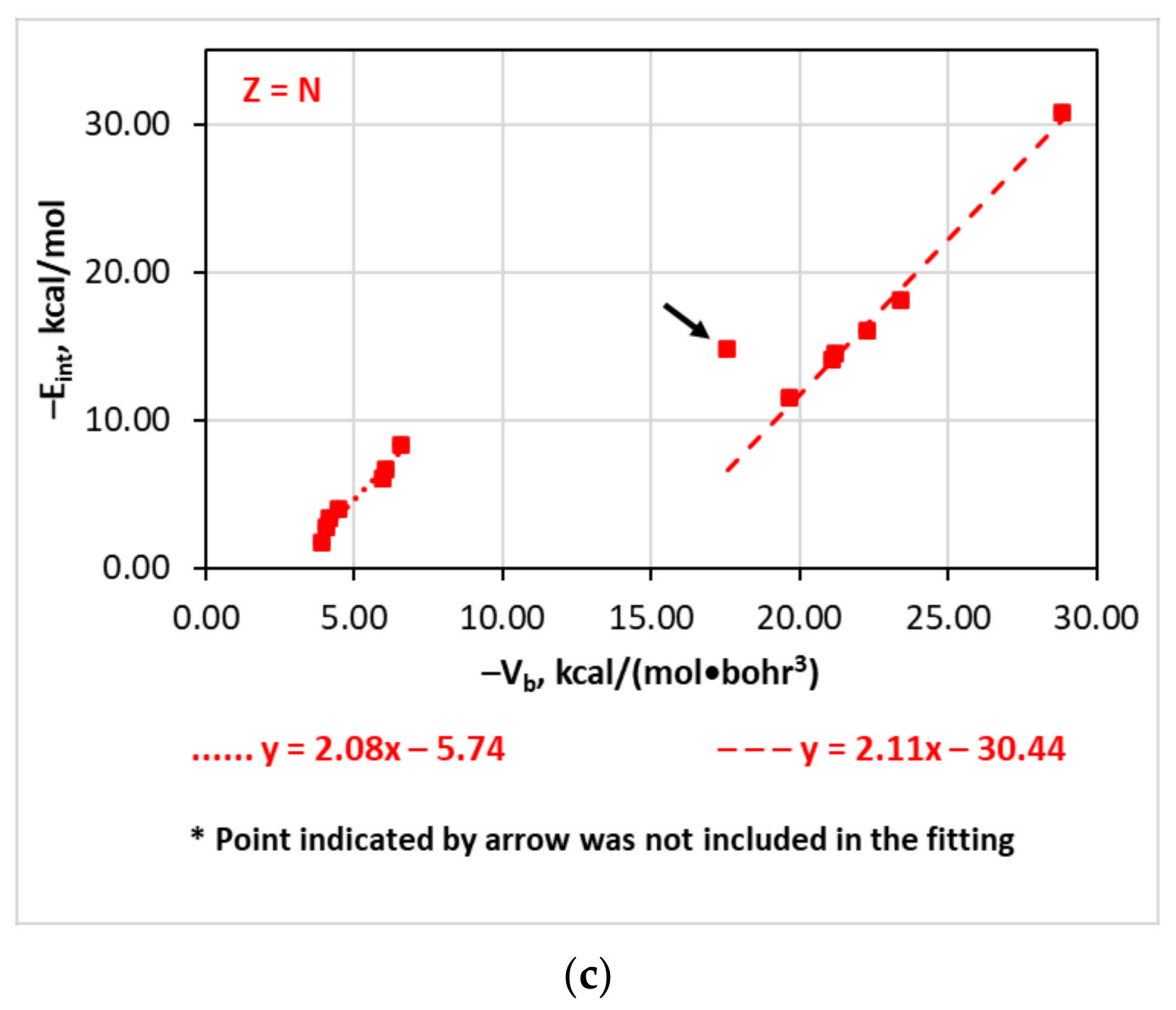
Plots of −E_int_ against −V_b_ for the [(A)*_n_*Z–Cl···I]^−^ structures; Z = Si + B and O + N + S + Hal (**a**), Z = C, P, Si, B, H (**b**), and Z = N (**c**).

As was mentioned above, the E_int_–property relationships for the [(A)*_n_*Z–Cl···I]^−^ series have a peculiar character. The more detailed analysis shows that such behavior is due to the tremendous effect of the second-order atom Z and the third-order groups A. First, the E_int_ values are low and do not exceed 14 kcal/mol when Z = C, P, Si, B, or H (Figure 7). The structures with Z = Hal have a relatively high E_int_ (28.95–45.43 kcal/mol) and those with Z = O, N, or S have a significant span of E_int_ (15.26–42.19, 1.70–30.79, and 5.47–87.68 kcal/mol, respectively).

Second, the E_int_(V_b_) trend slope for the combined series [(A)*_n_*Z–Cl···I]^−^ (Z = Si + B) is 6.75 while the slope of the linear correlation for the series [(A)*_n_*Z–Cl···I]^−^ (Z = O + N + S + Hal) is 1.43 (Figure 7). Dependences for the structures [(A)*_n_*Z–Cl···I]^−^ (Z = P, C) are not linear but exponential. The tangent slopes at the lowest energy points are 9.2 and 8.7.

Third, the [(A)*_n_*N–Cl···I]^−^ structures could be divided into two distinct groups (Figure 7), i.e., structures with a weaker Cl···I interaction (E_int_ < 10 kcal/mol, aliphatic and monoaryl amines, methylidene imine and nitroso compounds, Group I) and those with a stronger Cl···I interaction (E_int_ > 10 kcal/mol, diarylamines, difluoro(methylidene) imine and nitro compounds, Group II). The trend slope for these two groups is similar (2.1) but the intercept is very different (−5.7 and −30.4, respectively). The different behavior of these two groups is apparently associated with a different nature of the (A)*_n_*N part of the molecule. In Group I, (A)*_n_*N exhibits either electron donor or weak electron acceptor properties. In Group II, (A)*_n_*N is a strong electron acceptor. Furthermore, the structures of Group II have more extended conjugation systems compared to those of Group I. All these relationships considered together provide such a peculiar trend for the whole [(A)*_n_*Z–Cl···I]^−^ series. Interestingly, this effect of Z and A is much smaller for other series making possible the existence of linear correlations. Similar behavior was also found for the G_b_ and ρ_b_ estimators.

The great effect of the Z atom and A group on E_int_ does not permit an approximation of the latter via V_b_, G_b_, or ρ_b_ for the whole [(A)*_n_*Z–Cl···I]^−^ series. However, for the more narrow series [(A)*_n_*Si,B–Cl···I]^−^, [(A)*_n_*C–Cl···I]^−^, and [(A)*_n_*P–Cl···I]^−^, these relationships are of a good quality (R^2^ = 0.92–0.98, MAD = 0.28–0.77 kcal/mol) and they may be useful for practical applications. The d(Y…X) estimator is much better for the description of the [(A)*_n_*Z–Cl···I]^−^ series because it permits the reasonable approximation of E_int_ by a single two-exponent function.

## 5. Discussion

The estimate of E_int_ for the condensed phases is a much more difficult task than that for the gas phase. Both periodic solid state and cluster approaches often do not allow direct estimates of E_int_ as an energy difference because fragments may have multiple mutual weak intermolecular interactions. When it is possible, the adequate computational model is usually exceedingly large. For instance, accurate calculations of E_int_ for the A···B bond in a solid-state structure within the cluster approach requires explicit consideration of all intermolecular interactions which molecules A and B are involved in. An example of an adequate cluster for the direct estimates of E_int_ between the simple fragments I–C_4_F_8_–I and Cl^−^ in the X-ray structure ACIPOU is shown in Figure S7 and it has 386 atoms (this model is composed of the binuclear cluster I–C_4_F_8_–I···Cl^−^ surrounded by all molecules which form short contacts with the I–C_4_F_8_–I or Cl^−^ fragments).

Considering this situation, the practice of utilization of small bimolecular clusters which include only two molecules forming a contact of interest becomes increasingly popular. In other words, there are multiple attempts to approximate the properties of solid-state structures by gas phase calculations.

There are two points which invalidate this practice. First, such a bimolecular model ignores other intermolecular interactions in which both considered molecules participate. These secondary interactions may significantly affect the energy of the contact under study. For instance, E_int_ calculated for the I–CF_2_CF_2_–I···Cl^−^ halogen bond in the X-ray structure ACIPIO using the bimolecular approach is 20.07 kcal/mol. However, when four molecules are considered (three I–CF_2_CF_2_–I and one Cl^−^ ion, Figure S8), E_int_ for the I···Cl bond becomes 14.75 kcal/mol (see caption to Figure S8 for details) (this structure was taken as one of the examples bearing a short contact between the I atom and the Cl^−^ ion and where a relatively small cluster may be selected for reliable calculations of the interaction energy).

The second point is that geometry of bimolecular clusters is usually not optimized but taken from the X-ray structures since optimization often leads to the collapse of the structure compared to X-ray. In such calculations, the geometry considered is not equilibrium and does not belong to a minimum energy path for either adiabatic or vertical bond cleavage, and, hence, the resulting E_int_ has little physical meaning.

Application of the correlations established in this work for the rapid estimates of E_int_ in the condensed phase is free from the difficulties discussed above since it requires a single parameter (electron density or halogen bond length) directly obtained from experiment.

## 6. Final Remarks

In this work, correlations between the halogen bond interaction energy and experimentally accessible properties, i.e., electron density and bond length, were established for the series of structures [(A)*_n_*Z–I···Hal]^−^ and [(A)*_n_*Z–Hal···I]^−^ bearing the iodine atom (412 structures in total). Thus, together with the results published previously [116], this work finalizes the analysis of halogen bonds formed by a halide anion. Obtained correlations are of practical use for rapid estimates of the halogen bond energy in systems with a network of multiple weak interactions. Several concluding remarks and generalizations can be made.

First, among all structures [(A)*_n_*Z–Y···X]^−^, those with X = F demonstrate quite poor approximations of E_int_ with MAD of 3.29–4.76 kcal/mol. For other structures, except [(A)*_n_*Z–Cl···I]^−^, more reasonable correlations were obtained (MAD = 2.06–3.01 kcal/mol). Meanwhile, the approximations of E_int_ for halogen bonds formed by halide anions are worse than those obtained for homohalogen bonds formed by two neutral fragments [117].

Second, the most difficult case is the structures of the [(A)*_n_*Z–Cl···I]^−^ type. In fact, only two parameters were found to be able to adequately estimate E_int_ for this bond, i.e., H_b_ (for strong bonds with negative H_b_ values) and d_Y…X_.

Third, there is no unique equation to approximate E_int_ for all structures [(A)*_n_*Z–Y···X]^−^. For the electron density-based properties, the highest slopes (linear correlations) or curvatures (non-linear correlations) were found for the bonds formed by heavy halogens (I and Br) while the lowest slopes and curvatures were observed for X = F.

Fourth, the correlations derived for halogen bonds with halide anions are very different from those established for some hydrogen bonds and halogen bonds formed by two neutral fragments (Table 1). This and the previous [116] works indicate that each type of bonding requires the application of its own relationship.

Fifth, the ρ_b_, V_b_, G_b_, and d_Y···X_ estimators behave similarly. In contrast, the ∇^2^ρ_b_, λ_||,b_, and H_b_ parameters have limited significance as E_int_ estimators and they may be used only for some series or a certain range of the E_int_ values.

The relationships recommended for practical use to estimate E_int_ of the structures [(A)*_n_*Z–Y···I]^−^ and [(A)*_n_*Z–I···X]^−^ are given in Table 5.

## Figures and Tables

**Table 1:** Relationships between interaction energy (E_int_, kcal/mol) and potential or kinetic energy density (V_b_ or G_b_, kcal/(mol•bohr^3^)).

Type of Interaction	Estimator	Relationship	Reference
H···O	V_b_	E_int_ ~ 0.5V_b_	[82]
FH···FR	G_b_	E_int_ ~ −0.429G_b_	[83,86]
	V_b_	E_int_ ~ 0.37V_b_ − 3.1	[83]
Cl···X ^a^	V_b_	E_int_ ~ 0.49V_b_	[119]
	G_b_	E_int_ ~ −0.47G_b_	[119]
Br···X ^a^	V_b_	E_int_ ~ 0.58V_b_	[119]
	V_b_	E_int_ ~ 0.375V_b_ − 0.57	[120]
	G_b_	E_int_ ~ −0.57G_b_	[119]
I···X ^a^	V_b_	E_int_ ~ 0.68V_b_	[119]
	V_b_	E_int_ ~ 0.556V_b_ + 0.64	[120]
	G_b_	E_int_ ~ −0.67G_b_	[119]
F···F	G_b_	E_int_ ~ −0.129G_b_	[87]
Cl···Cl	V_b_	E_int_ ~ −0.1006V_b_^2^ − 0.218V_b_ − 0.55	[117]
	G_b_	E_int_ ~ −0.0841G_b_^2^ + 0.367G_b_ − 0.84	[117]
Br···Br	V_b_	E_int_ ~ −0.0926V_b_^2^ − 0.173V_b_ − 0.16	[117]
	G_b_	E_int_ ~ −0.1178G_b_^2^ + 0.73G_b_ − 1.5	[117]
I···I	V_b_	E_int_ ~ −0.0635V_b_^2^ − 0.217V_b_ − 0.25	[117]
	G_b_	E_int_ ~ −0.1564G_b_^2^ + 1.138G_b_ − 2.25	[117]

^a^ X = N, S, O, C in [119], X = O, N, F in [120].

**Table 2:** Enthalpies of formation of hydrogen and halogen bonds (in kcal/mol).

Structure	Method	ΔH ^a^ CP/no-CP ^b^
[H_3_C–H···F]^−^	M06-2X/6-31+G*	−6.8/−7.1
	CCSD/6-31+G*	−4.2/−5.1
	CCSD(T)//CCSD/6-31+G* ^c^	−4.3/−5.4
	CCSD/6-311+G**	−4.7/−5.8
	CCSD/aug-cc-pVTZ//6-31+G* ^d^	−6.0/−6.4
	CCSD(T)/aug-cc-pVTZ//CCSD/6-31+G* ^e^	−6.3/−6.8
	exp. ^f^	−6.7 ± 0.2
[F–H–F]^−^	M06-2X/6-31+G*	−50.2/−50.7
	CCSD/6-31+G*	−42.7/−44.8
	CCSD(T)//CCSD/6-31+G* ^c^	−42.8/−45.2
	CCSD/6-311+G**	−42.3/−46.5
	CCSD/aug-cc-pVTZ//6-31+G* ^d^	−43.7/−45.1
	CCSD(T)/aug-cc-pVTZ//CCSD/6-31+G* ^e^	−43.9/−45.4
	CCSD/aug-cc-pVTZ	−44.8/−46.3
	CCSD(T)//CCSD/aug-cc-pVTZ ^c^	−45.0/−46.6
	exp. ^g^	−45.8 ± 1.6
[HO–H···Cl]^−^	M06-2X/6-31+G*	−15.0/−15.2
	CCSD/6-31+G*	−12.3/−14.2
	CCSD(T)//CCSD/6-31+G* ^c^	−12.5/−14.5
	CCSD/aug-cc-pVTZ//6-31+G* ^d^	−13.3/−13.9
	CCSD(T)/aug-cc-pVTZ//CCSD/6-31+G* ^e^	−13.8/−14.4
	CCSD/aug-cc-pVTZ	−13.5/−14.2
	CCSD(T)//CCSD/aug-cc-pVTZ ^c^	−14.0/−14.8
	exp. ^h^	−14.9 ± 0.2
[HO–H···I]^−^	M06-2X/ADZP–DKH//ADZP ^i^	−10.3/−14.0
	CCSD(T)/aug-cc-pVTZ(PP)//CCSD/ADZP ^j^	−8.1/−11.0
	exp. ^h^	−11.1 ± 0.1
[H_3_C–H···I]^−^	M06-2X/ADZP–DKH//ADZP ^i^	−1.6/−3.1
	CCSD(T)/aug-cc-pVTZ(PP)//CCSD/ADZP ^j^	−0.4/−2.4
	exp. ^f^	−2.6 ± 0.2
[I–I–I]^−^	M06-2X/ADZP–DKH//ADZP ^k^	−29.3/−36.6
	CCSD(T)/aug-cc-pVTZ(PP)//CCSD/ADZP ^k^	−27.3/−30.6
	exp. ^l,m^	−30.1 ± 1.4

^a^ The calculated values are adiabatic. ^b^ CP/no-CP means with/without CP correction. ^c^ ΔH = ΔE(CCSD(T)) − ΔE(CCSD) + ΔH(CCSD). ^d^ ΔH = ΔE(CCSD/aug-cc-pVTZ) − ΔE(CCSD/6-31+G*) + ΔH(CCSD/6-31+G*). ^e^ ΔH = ΔE(CCSD(T)/aug-cc-pVTZ) − ΔE(CCSD/6-31+G*) + ΔH(CCSD/6-31+G*). ^f^ Ref. [134]. ^g^ Ref. [135]. ^h^ Ref. [136]. ^i^ ΔH = ΔE(M06-2X/ADZP-DKH) − ΔE(M06-2X/ADZP) + ΔH(M06-2X/ADZP). ^j^ ΔH = ΔE(CCSD(T)/aug-cc-pVTZ(PP)) − ΔE(CCSD/ADZP) + ΔH(CCSD/ADZP). ^k^ Adiabatic interaction energy in terms of E_int_. ^l^ Ref. [137]. ^m^ Enthalpy at 0 K.

**Table 3:** Theoretical and experimental electron densities (in e/A^3^) at the corresponding BCP.

Ref. Code	Contact	ρ_b,theor_ (M06-2X)	ρ_b,theor_ (PBE0-D3BJ)	ρ_b,exp_ [Ref.]
ICSD 194468	I···I	0.097	0.102	0.101 [139]
ETUDUT01	Cl···Cl	0.052	0.050	0.048(2) [140]
IJIGOU	Cl···Cl	0.033	0.032	0.03 [141]
FUFNOJ02	Cl···Cl	0.053	0.051	0.05 [141]
IJIHAL	Cl···Cl	0.045	0.044	0.03 [141]
CIHBAX01	Br···Cl	0.079	0.078	0.081(2) [142]
BZQDCL11	Cl···O	0.055	0.054	0.054(1) [143]
PCPHOL01	Cl···Cl	0.050	0.048	0.058 [144]
ROFKAZ01	Br···Br	0.061	0.060	0.063 [144]
XIPRUL	I···N	0.174	0.176	0.154(12) [145]
XIPRUL	I···O	0.099	0.105	0.092(7) [145]

**Table 4:** The R^2^ and MAD (kcal/mol) values calculated for the V_b_, G_b_, ρ_b_, and d(Y···X) estimators for the series [(A)*_n_*Z–Y···X]^−^.

Series	V_b_		G_b_		ρ_b_		d(Y···X)	
	R^2^	MAD	R^2^	MAD	R^2^	MAD	R^2^	MAD
[(A)*_n_*Z–I···F]^−^	0.91	4.76	0.92	4.66	0.91	4.68	0.91	4.77
[(A)*_n_*Z–Cl···F]^−^^a^	0.95	3.36	0.95	3.53	0.94 ^b^	3.59 ^b^	0.94	3.88
[(A)*_n_*Z–Br···F]^−^^a^	0.94	3.83	0.95	3.29	0.94 ^b^	3.73 ^b^	0.93	4.00
[(A)*_n_*Z–I···Cl]^−^	0.95	2.43	0.96	2.34	0.95	2.49	0.95	2.55
[(A)*_n_*Z–Cl···Cl]^−^^a^	0.95	2.06	0.95	2.08	0.95	2.15	0.95	2.17
[(A)*_n_*Z–Br···Cl]^−^^a^	0.94	2.73	0.94	2.63	0.93	3.01	0.94	2.78
[(A)*_n_*Z–Cl···Br]^−^^a^	0.96	2.17	0.95	2.31	0.95	2.35	0.96	2.33
[(A)*_n_*Z–Br···Br]^−^^a^	0.94	2.75	0.94	2.77	0.93	3.01	0.93	2.89
[(A)*_n_*Z–I···Br]^−^	0.95	2.22	0.96	2.17	0.95	2.23	0.95	2.29
[(A)*_n_*Z–I···I]^−^	0.96	2.34	0.95	2.48	0.96	2.21	0.96	2.27
[(A)*_n_*Z–Br···I]^−^	0.95	2.70	0.95	2.66	0.96	2.41	0.96	2.50

^a^ From [116]. ^b^ The data are for the binominal fittings.

**Table 5:** Relationships recommended for the estimates of E_int_ (in kcal/mol) for the structures [(A)*_n_*Z–Y···I]^−^ and [(A)*_n_*Z–I···X]^−^ at the M06-2X/ADZP–DKH level of theory (V_b_, G_b_, and H_b_ in kcal/(mol•bohr^3^), ρ_b_ in e/Å^3^, λ_||,b_ in e/Å^5^, d_Y···X_ in Å).

Series	Estimator	Equation	R^2^	MAD
[(A)*_n_*Z–I···F]^−^	V_b_	−E_int_ = −0.77V_b_ − 6.50	0.91	4.76
	G_b_	−E_int_ = 1.04G_b_ − 8.97	0.92	4.66
	ρ_b_	−E_int_ = 150.4ρ_b_^2^ − 30.29ρ_b_ + 12.69	0.91	4.68
	d_Y···X_	−E_int_ = 5.28 × 10^4^e^−3.327d^	0.91	4.77
[(A)*_n_*Z–I···Cl]^−^	V_b_	−E_int_ = −1.46V_b_ − 1.65	0.95	2.43
	G_b_	−E_int_ = 0.121G_b_^2^ − 0.57G_b_ + 5.77	0.96	2.34
	H_b_	−E_int_ = −2.75H_b_ + 14.80 ^a^	0.95	2.43
	ρ_b_	−E_int_ = 145.6ρ_b_^2^ + 46.09ρ_b_ + 1.50	0.95	2.49
	λ_||,b_	−E_int_ = 3.69e^0.518λ||,b^ + 2 × 10^−4^e^3.054λ||,b^	0.95	2.51
	d_Y···X_	−E_int_ = 4.28 × 10^4^e^−2.650d^	0.95	2.55
[(A)*_n_*Z–I···Br]^−^	V_b_	−E_int_ = −2.12V_b_ − 3.88	0.95	2.22
	G_b_	−E_int_ = 0.212G_b_^2^ − 0.77G_b_ + 4.88	0.96	2.17
	H_b_	−E_int_ = −4.45H_b_ + 14.52 ^a^	0.95	2.08
	ρ_b_	−E_int_ = 197.6ρ_b_^2^ + 56.42ρ_b_ + 0.11	0.95	2.23
	λ_||,b_	−E_int_ = 2.51e^0.867λ||,b^ + 1.3 × 10^−8^e^6.390λ||,b^	0.96	2.06
	d_Y···X_	−E_int_ = 7.21 × 10^4^e^−2.688d^	0.95	2.29
[(A)*_n_*Z–I···I]^−^	V_b_	−E_int_ = −2.34V_b_ − 2.56	0.96	2.34
	G_b_	−E_int_ = 0.360G_b_^2^ − 2.05G_b_ + 7.87	0.95	2.48
	H_b_	−E_int_ = −4.38H_b_ + 16.60 ^a^	0.96	2.07
	ρ_b_	−E_int_ = 232.2ρ_b_^2^ + 54.11ρ_b_ + 0.49	0.96	2.21
	d_Y···X_	−E_int_ = 1.30 × 10^5^e^−2.746d^	0.96	2.27
[(A)*_n_*Z–Cl···I]^−^	H_b_	−E_int_ = 0.0513H_b_^2^ − 1.41H_b_ + 9.60 ^a^	0.95	3.33
	d_Y···X_	−E_int_ = 1.4 × 10^10^e^−8.078d^ + 2.21 × 10^3^e^−1.834d^	0.96	2.70
[(A)*_n_*Z–Br···I]^−^	V_b_	−E_int_ = −1.95V_b_ − 1.92	0.95	2.70
	G_b_	−E_int_ = 0.248G_b_^2^ − 1.39G_b_ + 6.48	0.95	2.66
	H_b_	−E_int_ = −3.74H_b_ + 14.81 ^a^	0.96	2.31
	ρ_b_	−E_int_ = 235.6ρ_b_^2^ + 13.34ρ_b_ + 2.68	0.96	2.41
	d_Y···X_	−E_int_ = 1.38 × 10^5^e^−2.957d^	0.96	2.50
[(A)*_n_*P–Cl···I]^−^	V_b_	−E_int_ = 14.92 − 68.71e^0.639Vb^	0.97	0.64
[(A)*_n_*C–Cl···I]^−^	V_b_	−E_int_ = 9.84 − 57.41e^0.725Vb^	0.92	0.77
[(A)*_n_*Si,B–Cl···I]^−^	V_b_	−E_int_ = −6.75V_b_ − 12.33	0.98	0.28

^a^ For E_int_ > 10 kcal/mol.

## Data Availability

The data presented in this study are available in supplementary material and from the author upon request.

## Supplementary Materials

The following are available online: Figure S1: Correlations between theoretical and experimental values of electron density; Figure S2: Correlations between −E_int_ and −V_b_ calculated at the M06-2X/ADZP–DKH and PBE0-D3BJ/ADZP–DKH levels of theory; Figure S3: Plot of the slope of the E_int_(V_b_) dependence against the difference of Pauling’s electronegativities of the atoms X and Y; Figures S4–S6: Plots of −E_int_ against ∇^2^_ρb_, λ_||,b_, and H_b_; Figure S7: Example of an adequate cluster for the direct estimates of E_int_ between the fragments I–C_4_F_8_–I and Cl^−^ in the X-ray structure ACIPOU; Figure S8: Four-molecule computational model of the structure ACIPIO; Table S1: Relationships between interaction energy and electron density-based estimators, Table S2: Calculated structures.
