# Supplementary material for: Strength of the [Z–I···Hal]− and [Z–Hal···I]− Halogen Bonds: Electron Density Properties and Halogen Bond Length as Estimators of Interaction Energy
Source: Molecules. 2021 Apr 5;26(7):2083. doi: 10.3390/molecules26072083 (PMC8038634; doi:10.3390/molecules26072083)
Supplement: Supplementary file 1 [file molecules-26-02083-s001.pdf]

Strength of the $[Z–I\cdots Hal]^-$ and $[Z–Hal\cdots I]^-$ halogen bonds: electron density properties and halogen bond length as estimators of interaction energy

Maxim L. Kuznetsov [1,2,*]

[1] Centro de Química Estrutural, Instituto Superior Técnico, Universidade de Lisboa, Avenida Rovisco Pais, 1049-001 Lisbon, Portugal; max@mail.ist.utl.pt
[2] Institute of Chemistry, Saint Petersburg State University, Universitetskaya Nab. 7/9, 199034, Saint Petersburg, Russian Federation.
* Correspondence: max@mail.ist.utl.pt; Tel.: +351-218-419-236

Supplementary Material

Table S1. Relationships between interaction energy (E_{int}, kcal/mol) and electron density based estimators.

Type of interaction	Estimator	Relationship	Reference
H…O	V_b	$E_{int} \sim 0.5V_b$	[81]
FH…FR	G_b	$E_{int} \sim -0.429G_b$	[82,85]
	V_b	$E_{int} \sim 0.37V_b - 3.1$	[82]
	ρ_b	$E_{int} \sim -186\rho_b + 2.3$	[82]
	$\nabla^2\rho_b$	$E_{int} \sim -2.52\nabla^2\rho_b - 5.2$	[82]
	$\lambda_{\|\|,b}$	$E_{int} \sim -6.7\lambda_{\|\|,b} + 2.3$	[82]
Cl…X[b]	V_b	$E_{int} \sim 0.49V_b$	[118]
	G_b	$E_{int} \sim -0.47G_b$	[118]
Br…X[b]	V_b	$E_{int} \sim 0.58V_b$	[118]
	V_b	$E_{int} \sim 0.375V_b - 0.57$	[119]
	G_b	$E_{int} \sim -0.57G_b$	[118]
I…X[b]	V_b	$E_{int} \sim 0.68V_b$	[118]
	V_b	$E_{int} \sim 0.556V_b + 0.64$	[119]
	G_b	$E_{int} \sim -0.67G_b$	[118]
F⋯F	G_b	$E_{int} \sim -0.129G_b$	[86]
Cl…Cl	V_b	$E_{int} \sim -0.1006V_b^2 - 0.218V_b - 0.55$	[116]
	G_b	$E_{int} \sim -0.0841G_b^2 + 0.367G_b - 0.84$	[116]
	ρ_b	$E_{int} \sim -535.9\rho_b^2 + 31.13\rho_b - 0.87$	[116]
	$\nabla^2\rho_b$	$E_{int} \sim -0.09e^{2.405\nabla^2\rho_b} - 0.17$	[116]
	$\lambda_{\|\|,b}$	$E_{int} \sim -0.099e^{1.762\lambda_{\|\|,b}} - 0.17$	[116]
Br…Br	V_b	$E_{int} \sim -0.0926V_b^2 - 0.173V_b - 0.16$	[116]
	G_b	$E_{int} \sim -0.1178G_b^2 + 0.73G_b - 1.5$	[116]
	ρ_b	$E_{int} \sim -380.6\rho_b^2 + 24.78\rho_b - 0.42$	[116]
	$\nabla^2\rho_b$	$E_{int} \sim -0.07e^{2.624\nabla^2\rho_b} + 0.1$	[116]
	$\lambda_{\|\|,b}$	$E_{int} \sim -0.3e^{1.306\lambda_{\|\|,b}} + 0.71$	[116]
I…I	V_b	$E_{int} \sim -0.0635V_b^2 - 0.217V_b - 0.25$	[116]
	G_b	$E_{int} \sim -0.1564G_b^2 + 1.138G_b - 2.25$	[116]
	H_b	$E_{int} \sim 2.35H_b - 1.87$	[116]
	ρ_b	$E_{int} \sim -305.2\rho_b^2 + 21.78\rho_b - 0.44$	[116]
	$\lambda_{\|\|,b}$	$E_{int} \sim -0.031e^{2.818\lambda_{\|\|,b}} + 0.25$	[116]

[a] V_b, G_b, and H_b in kcal/(mol•bohr^3), ρ_b in e/Å^3, $\nabla^2\rho_b$ and $\lambda_{\|\|,b}$ in e/Å^5. [b] X = N, S, O, C in ref. [118], X = O, N, F in ref. [119].

Table S2. Calculated structures.

$[(A)_nZ–Y•••X]^–$ (Y = I, X = F, Cl, Br, I; Y = Cl, Br, X = I)			
**Z = Hal**<br>$(A)_nZ$ = F<br>Cl, Br<br>I<br>**Z = C (sp^3)**<br>$(A)_nZ$ = Me<br>$H_2(Me)C$–<br>$H(Me)_2C$–<br>Me_3C–<br>H_2FC–<br>$H(F)_2C$–<br>F_3C–<br>**Z = C (sp^2)**<br>$(A)_nZ$ = H_2C=CH–<br>H(O=)C–<br>Me(O=)C–<br>(MeO)(O=)C–<br>Ph<br>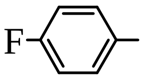<br>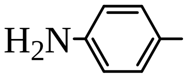<br>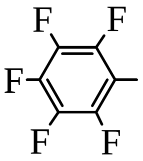<br>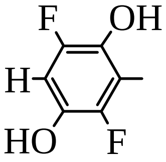<br>(a)	**Z = N**<br>$(A)_nZ$ = H_2N–<br>Me_2N–<br>H(Ph)N–<br>Ph_2N– (a)<br>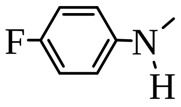<br>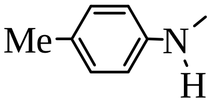<br>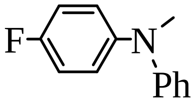 (a)<br>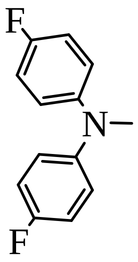 (a)<br>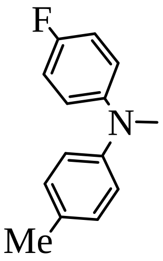 (a)<br>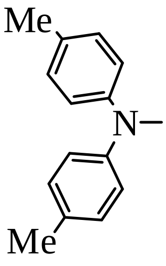 (a)	**Z = N**<br>$(A)_nZ$ = C=N–<br>H_2C=N–<br>F_2C=N–<br>Me_2C=N–<br>O_2N–<br>**Z = S**<br>$(A)_nZ$ =<br>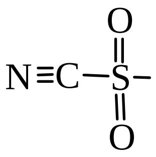<br>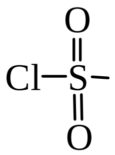<br>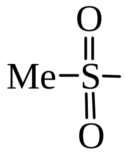<br>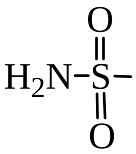<br>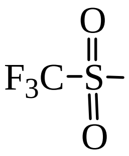<br>Me(O=)S–<br>F_3C(O=)S–<br>Cl(O=)S–<br>N≡C(O=)S–	**Z = P**<br>$(A)_nZ$ = H_2P–<br>H(F)P–<br>F_2P–<br>Cl_2(O=)P–<br>F_2(O=)P–<br>H_2(O=)P–<br>H(F)(O=)P–<br>Me_2(O=)P–<br>**Z = B**<br>$(A)_nZ$ = F_2B–<br>H_2B–<br>H(F)B–<br>H(HO)B–<br>**Z = Si**<br>$(A)_nZ$ = Me_3Si–<br>F_3Si–<br>H3Si–<br>$H_2(F)Si$–<br>$H(F)_2Si$–<br>**Z = O**<br>$(A)_nZ$ = HO–<br>MeO–<br>PhO–<br>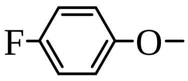<br>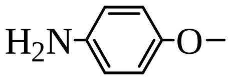<br>**Z = H**<br>$(A)_nZ$ = H
**Z = C (sp^2)**<br>$(A)_nZ$ = HC≡C–<br>MeC≡C–<br>FC≡C–			

(a) Except Y = X = I.

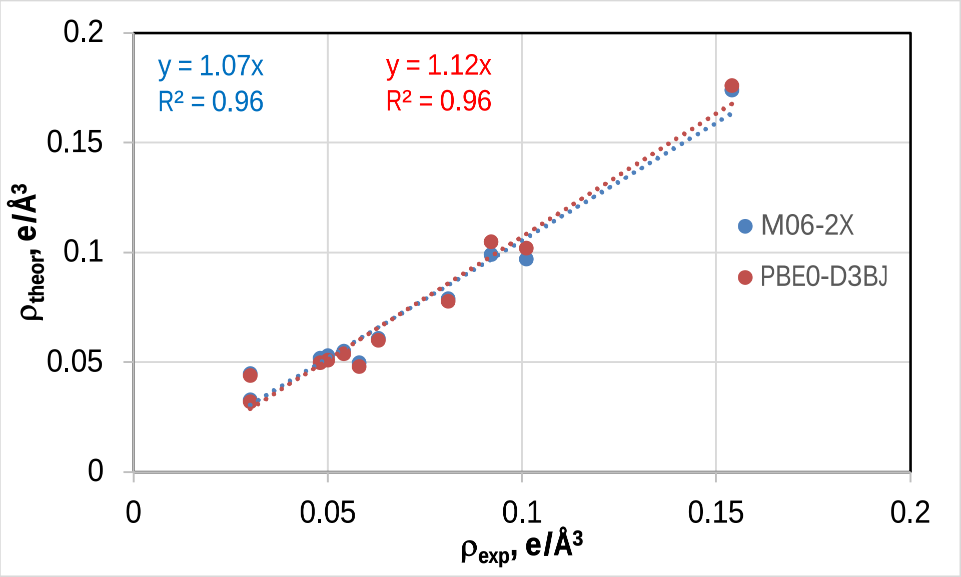

Figure S1. Correlations between theoretical and experimental values of the electron density (see Table 3 for details).

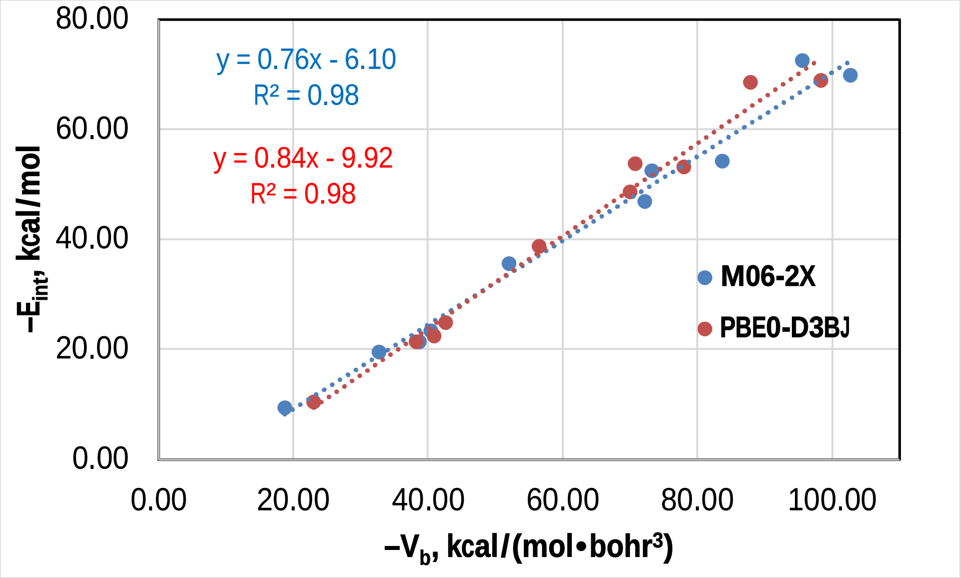

Figure S2. Correlations between $-E_{int}$ and $-V_b$ calculated at the M06-2X/ADZP-DKH and PBE0-D3BJ/ADZP-DKH levels of theory.

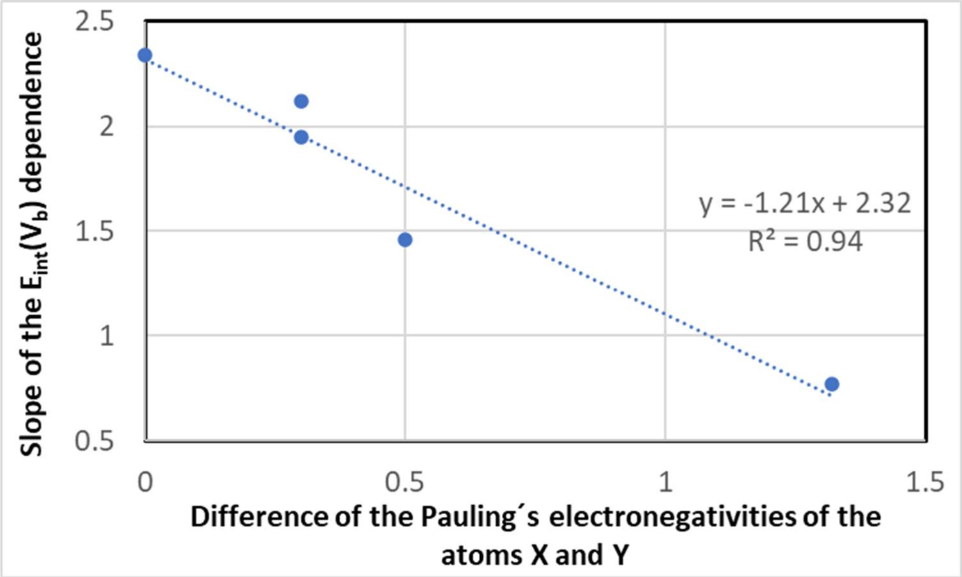

Figure S3. Plot of the slope of the $E_{int}(V_b)$ dependence against difference of the Pauling´s electronegativities of the atoms X and Y in structures $[(A)_nZ–I\cdots X]^–$ and $[(A)_nZ–Br\cdots I]^–$ (X = F, Cl, Br, I).

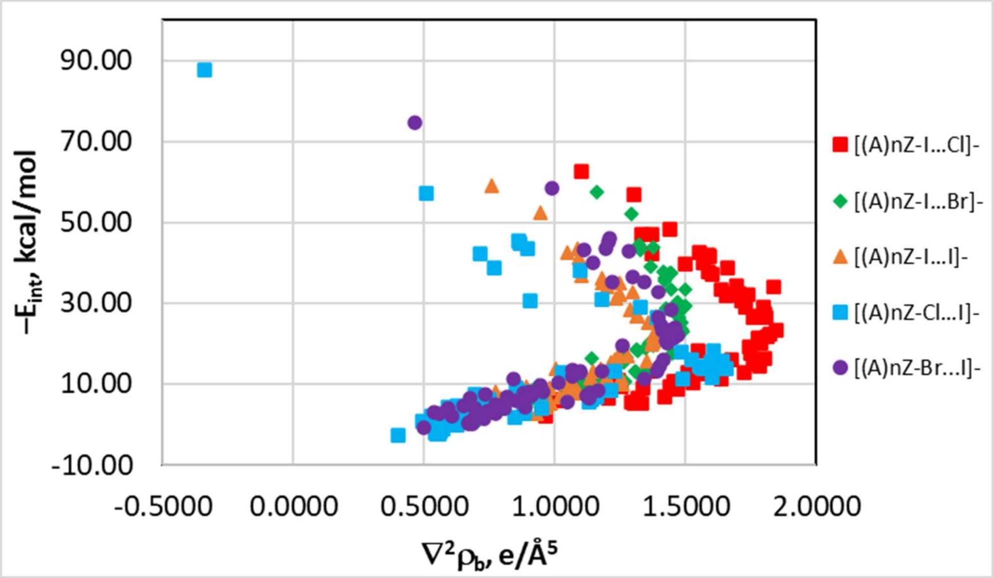

Figure S4. Plots of $–E_{int}$ *vs.* $\nabla^2\rho_b$.

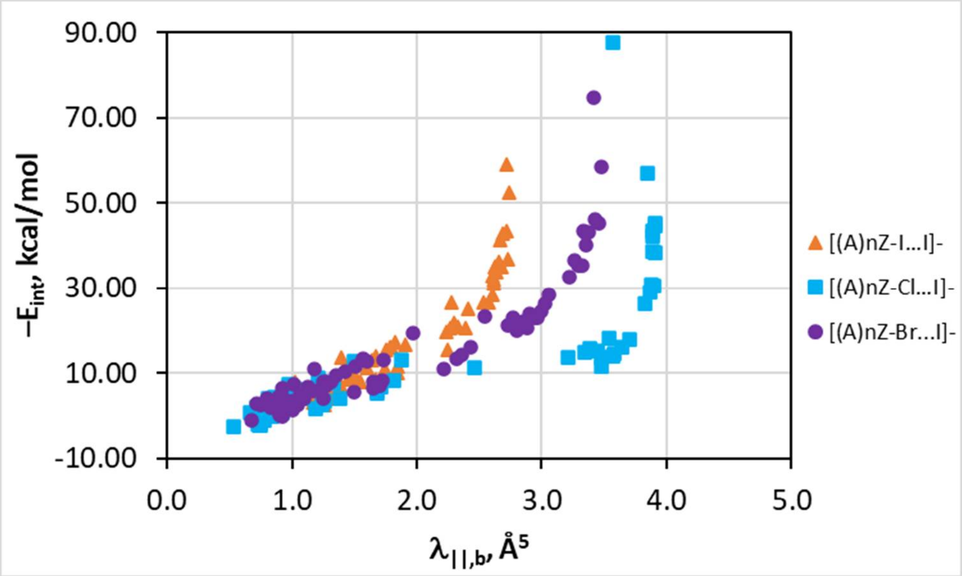

Figure S5. Plots of $–E_{int}$ *vs.* $\lambda_{\|,b}$.

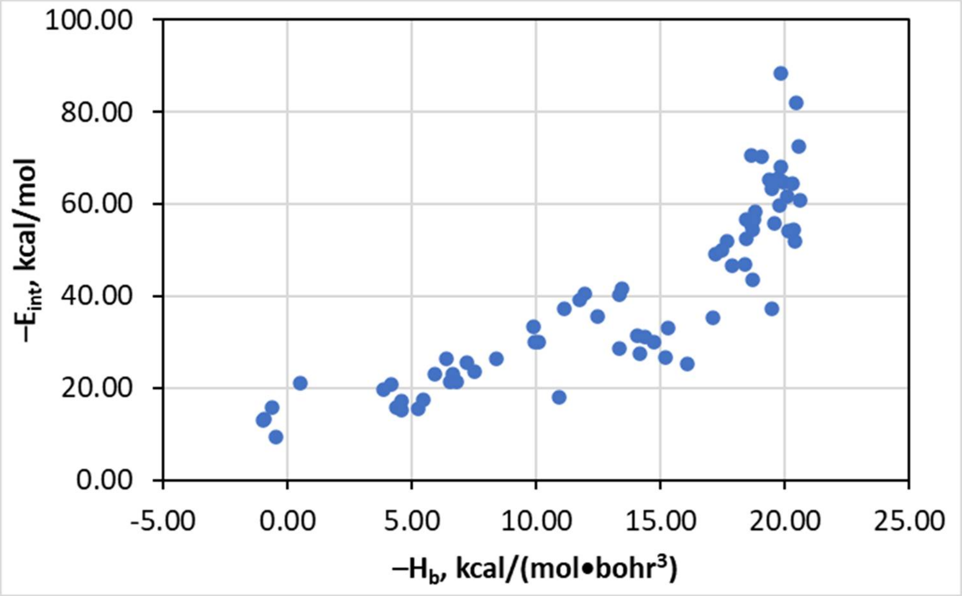

Figure S6. Plot of $–E_{int}$ *vs.* $–H_b$ for the $[(A)_nZ–I•••F]^–$ series.

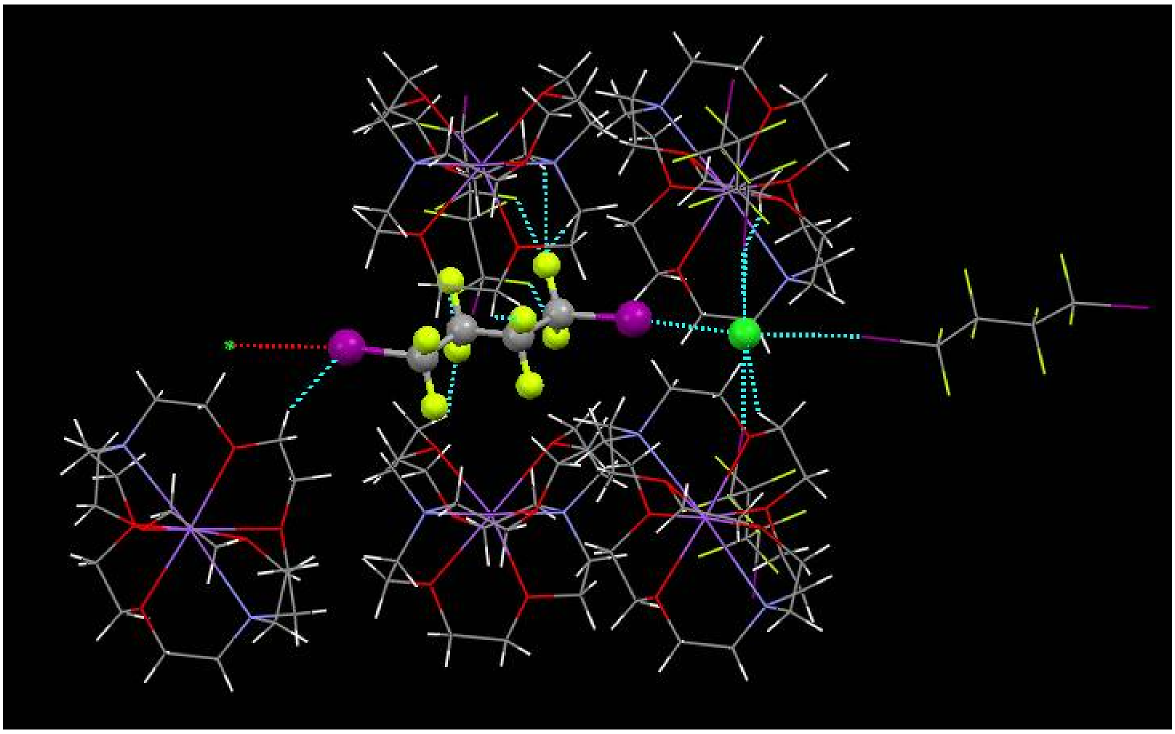

Figure S7. Example of the adequate cluster for the direct estimates of E_{int} between the fragments I–C_4F_8–I and Cl^- in the X-ray structure ACIPOU.

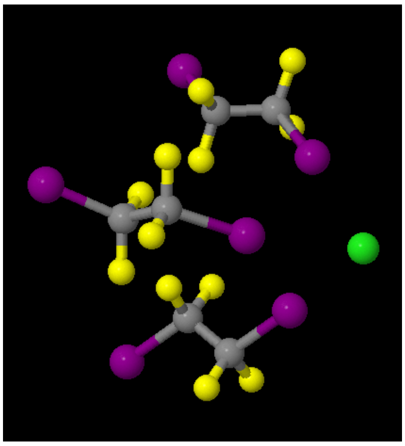

Figure S8. Four-molecule computational model of the structure ACIPIO (criterium for this model was inclusion of all ICF_2CF_2I fragments with the nearest neighbor I•••Cl distance in the three directions of 3.73 Å relatively to the given Cl^- ion. E_{int} for the individual I•••Cl contact was calculated as E_{int}(I•••Cl) = E_{int}(total)/3, where E_{int}(total) is the total interaction energy between $(ICF_2CF_2I)_3$ and Cl^-).
